# Supplementary material for: Uncovering Divergence in Gene Expression Regulation in the Adaptation of Yeast to Nitrogen Scarcity
Source: mSystems. 2021 Aug 24;6(4):e00466-21. doi: 10.1128/mSystems.00466-21 (PMC8407396; doi:10.1128/mSystems.00466-21)
Supplement: FIG S1 [file msystems.00466-21-sf001.pdf]

A

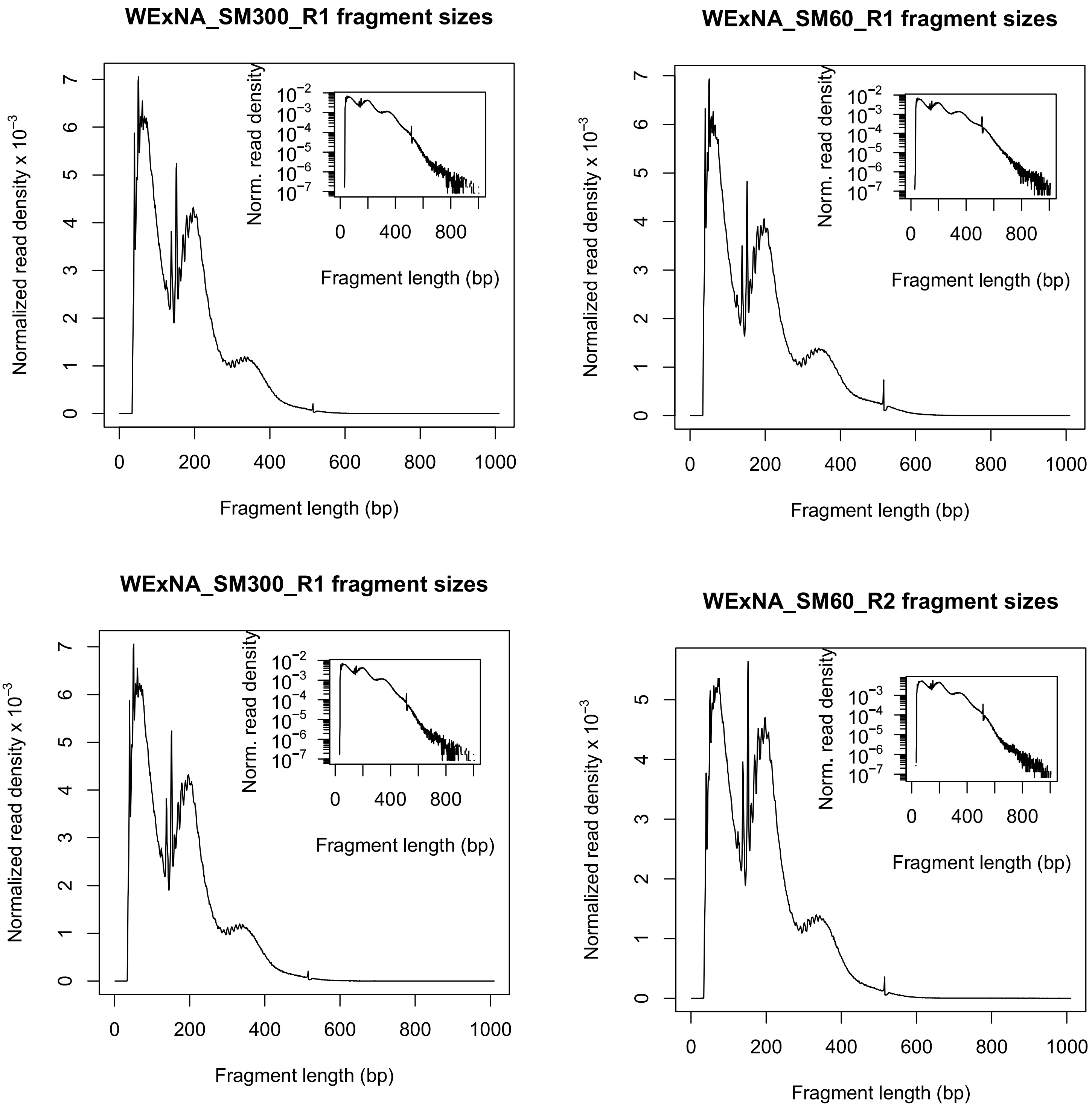

WExNA_SM300_R1 fragment sizes
WExNA_SM60_R1 fragment sizes
WExNA_SM300_R1 fragment sizes
WExNA_SM60_R2 fragment sizes
Normalized read density x 10^{-3}
Norm. read density
Fragment length (bp)

B

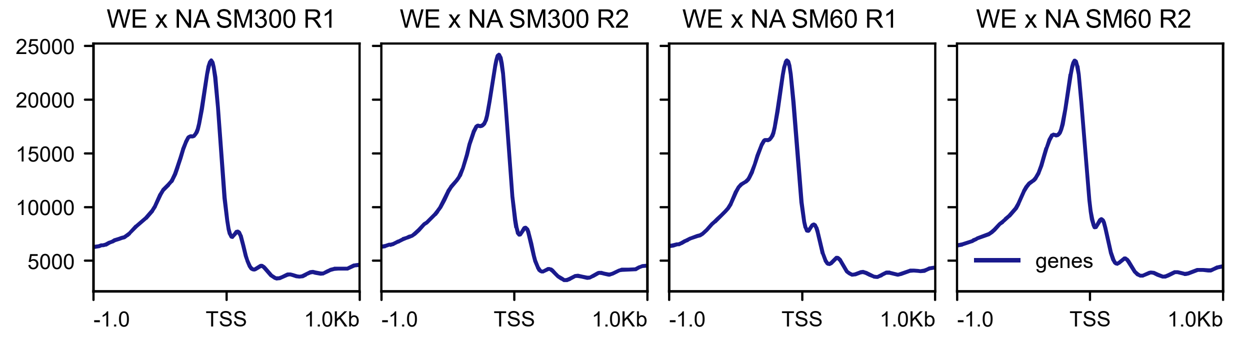

WE x NA SM300 R1
WE x NA SM300 R2
WE x NA SM60 R1
WE x NA SM60 R2
genes
-1.0
TSS
1.0Kb
