# Supplementary material for: Uncovering Divergence in Gene Expression Regulation in the Adaptation of Yeast to Nitrogen Scarcity
Source: mSystems. 2021 Aug 24;6(4):e00466-21. doi: 10.1128/mSystems.00466-21 (PMC8407396; doi:10.1128/mSystems.00466-21)
Supplement: FIG S2 [file msystems.00466-21-sf002.pdf]

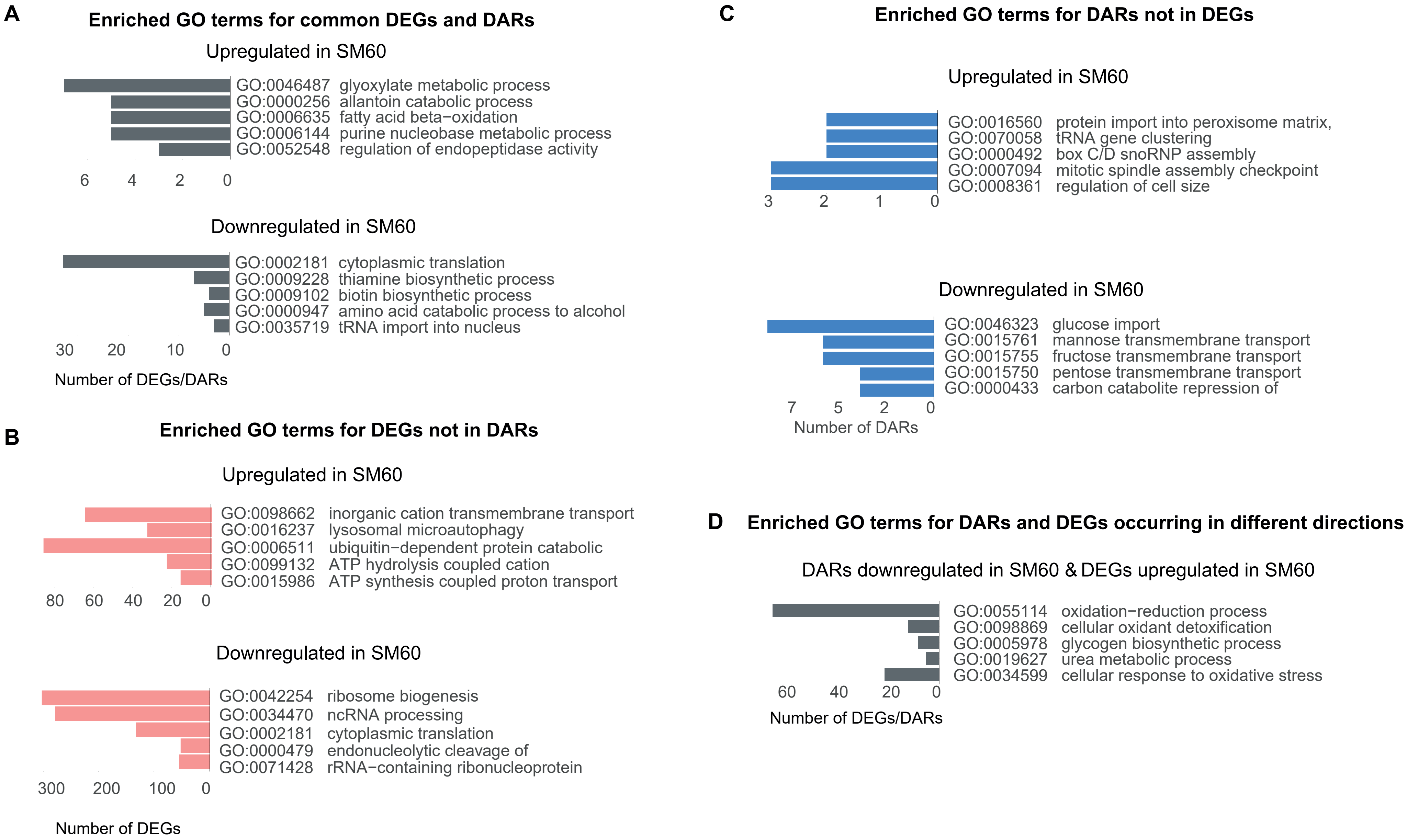
A
Enriched GO terms for common DEGs and DARs
Upregulated in SM60
GO:0046487 glyoxylate metabolic process
GO:0000256 allantoin catabolic process
GO:0006635 fatty acid beta−oxidation
GO:0006144 purine nucleobase metabolic process
GO:0052548 regulation of endopeptidase activity
6 4 2 0
Downregulated in SM60
GO:0002181 cytoplasmic translation
GO:0009228 thiamine biosynthetic process
GO:0009102 biotin biosynthetic process
GO:0000947 amino acid catabolic process to alcohol
GO:0035719 tRNA import into nucleus
30 20 10 0
Number of DEGs/DARs
B
Enriched GO terms for DEGs not in DARs
Upregulated in SM60
GO:0098662 inorganic cation transmembrane transport
GO:0016237 lysosomal microautophagy
GO:0006511 ubiquitin−dependent protein catabolic
GO:0099132 ATP hydrolysis coupled cation
GO:0015986 ATP synthesis coupled proton transport
80 60 40 20 0
Downregulated in SM60
GO:0042254 ribosome biogenesis
GO:0034470 ncRNA processing
GO:0002181 cytoplasmic translation
GO:0000479 endonucleolytic cleavage of
GO:0071428 rRNA−containing ribonucleoprotein
300 200 100 0
Number of DEGs
C
Enriched GO terms for DARs not in DEGs
Upregulated in SM60
GO:0016560 protein import into peroxisome matrix,
GO:0070058 tRNA gene clustering
GO:0000492 box C/D snoRNP assembly
GO:0007094 mitotic spindle assembly checkpoint
GO:0008361 regulation of cell size
3 2 1 0
Downregulated in SM60
GO:0046323 glucose import
GO:0015761 mannose transmembrane transport
GO:0015755 fructose transmembrane transport
GO:0015750 pentose transmembrane transport
GO:0000433 carbon catabolite repression of
7 5 2 0
Number of DARs
D
Enriched GO terms for DARs and DEGs occurring in different directions
DARs downregulated in SM60 & DEGs upregulated in SM60
GO:0055114 oxidation−reduction process
GO:0098869 cellular oxidant detoxification
GO:0005978 glycogen biosynthetic process
GO:0019627 urea metabolic process
GO:0034599 cellular response to oxidative stress
60 40 20 0
Number of DEGs/DARs
