# Supplementary figures and images for: Uncovering Divergence in Gene Expression Regulation in the Adaptation of Yeast to Nitrogen Scarcity
Source: mSystems. 2021 Aug 24;6(4):e00466-21. doi: 10.1128/mSystems.00466-21 (PMC8407396; doi:10.1128/mSystems.00466-21)

# A

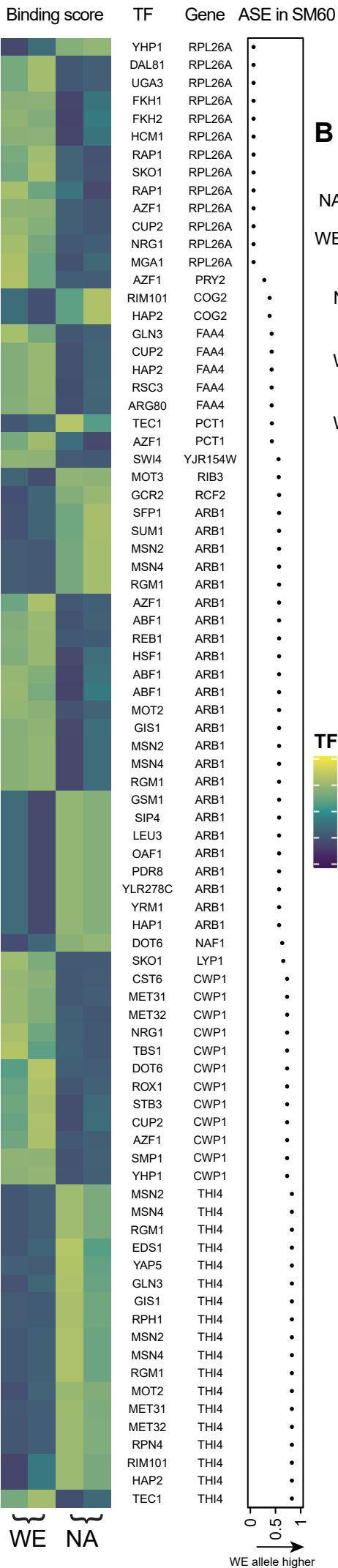

B

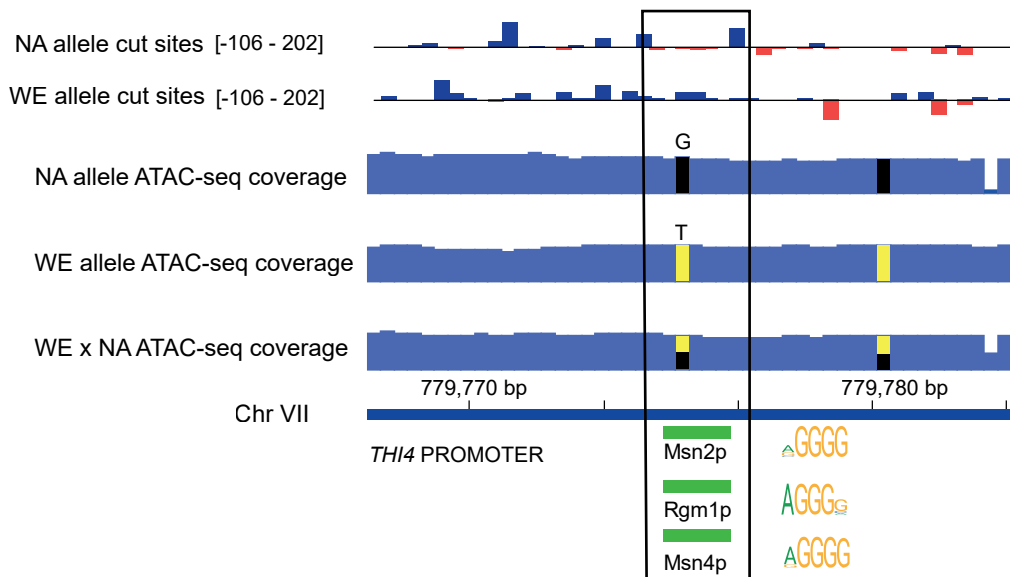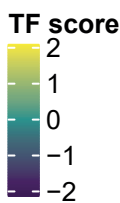

Supplement: FIG S3 [file msystems.00466-21-sf003.pdf]
